# Automated Cattle Head and Ear Pose Estimation Using Deep Learning for Animal Welfare Research

**DOI:** 10.3390/vetsci12070664

**Published:** 2025-07-13

**Authors:** Sueun Kim

**Affiliations:** Laboratory of Large Animal Clinical Medicine, Graduate School of Veterinary Sciences, Osaka Metropolitan University, Osaka 598-8531, Japan; kim73@omu.ac.jp

**Keywords:** cattle stress assessment, deep learning, non-invasive monitoring, pose estimation, animal behavior analysis

## Abstract

With the increasing importance of animal welfare, behavioral indicators such as changes in head and ear posture are widely recognized as non-invasive and field-applicable markers for evaluating the emotional state and stress levels of animals. However, traditional visual observation methods are often subjective, as assessments can vary between observers, and are unsuitable for long-term, quantitative monitoring. This study proposes an artificial intelligence (AI)-based system for the detection and pose estimation of cattle heads and ears using deep learning techniques. The system integrates Mask R-CNN for accurate object detection and FSA-Net for robust 3D pose estimation (yaw, pitch, and roll) of cattle heads and left ears. Comprehensive datasets were constructed from images of Japanese Black cattle, collected under natural conditions and annotated for both detection and pose estimation tasks. The proposed framework achieved mean average precision (mAP) values of 0.79 for head detection and 0.71 for left ear detection and mean absolute error (MAE) of approximately 8–9° for pose estimation, demonstrating reliable performance across diverse orientations. This approach enables long-term, quantitative, and objective monitoring of cattle behavior, offering significant advantages over traditional subjective stress assessment methods. The developed system holds promise for practical applications in animal welfare research and real-time farm management.

## Simple Summary

This study developed an AI system that uses deep learning (Mask R-CNN and FSA-Net) to automatically detect and estimate the 3D pose of cattle heads and ears from images, achieving high accuracy. The system enables non-invasive, real-time monitoring of cattle behavior, offering a practical alternative to invasive stress assessments. Designed for integration with farm cameras, it supports early stress detection and efficient management. Further research is needed to expand datasets, validate stress correlations, and optimize for mobile devices.

## 1. Introduction

As technology advances and interest in animal welfare grows, non-invasive methods are increasingly being utilized in the field [1]. In particular, behavioral indicators are widely used to evaluate stress and pain in a variety of animal species [2,3,4] due to their non-invasive nature and practical applicability. For example, in dairy cattle, behavioral indicators such as altered posture, facial expressions, and changes in ear and head positions provide valuable insights into pain and stress levels [4]. Analyzing the pose of the head and ears plays a crucial role in animal behavior research, as these features help assess an animal’s emotional state, stress, and pain. For example, under stress, animals tend to use their left eye more frequently, and the head is often turned to the right, allowing the animal to monitor threats with the left eye. This behavior is linked to brain lateralization, where the right brain hemisphere—connected to the left eye—coordinates fight-or-flight responses [5]. In cattle studies, this tendency is observed when animals encounter unfamiliar people (a novel stimulus and potential threat) [6] or when forced to pass through narrow pathways [7], both situations in which they show a preference for using their left eye. The position of the ears is also an important indicator of stress or pain. In cattle and horses, ears that are directed forward indicate little or no pain, ears pulled back indicate a certain degree of pain, and ears hanging down, like those of a lamb, indicate a more severe pain state [4,8]. However, because the direction of the head and ears can change rapidly or shift in multiple directions over short periods, it is difficult to obtain reliable data through visual observation alone. Therefore, long-term monitoring and quantitative analysis are essential.

Recent rapid advances in AI and computer vision technologies have opened new possibilities for animal behavior research. Deep learning-based object detection plays a crucial role in accurately identifying regions of interest within images or videos. Object detection is typically divided into two main approaches: one involves marking objects of interest with bounding boxes (e.g., YOLO [9], Faster R-CNN [10]), while the other uses image segmentation techniques to delineate object boundaries at the pixel level (e.g., Mask R-CNN [11]). When combined with infrared cameras, these object detection methods enable even more powerful analysis. For example, by detecting specific anatomical features such as the nostrils or eyes of animals, temperature data corresponding to those locations can be collected to assess breathing patterns [12] or changes in body temperature [13] in a non-contact manner. This approach is highly useful for real-time monitoring of animal stress, pain, and health status. In addition, animal pose estimation technology is widely used for analyzing animal movement and behavior. Animal pose estimation refers to the automatic identification of key body parts (such as the head, legs, and tail) in images or video and the quantitative analysis of posture and movement by connecting these points. A variety of open-source tools, including DeepLabCut [14], SLEAP [15], SuperAnimal [16], and LabGym [17], have been developed. These tools are applied to diverse animal models such as mice, rats, birds, and horses, making it easier for researchers to collect and analyze animal behavior data [14,15,16,17,18].

While animal pose estimation tracks the positions and movements of the entire body, head pose estimation is a more specialized technique that specifically analyzes the orientation of the head. In this context, “pose estimation” refers to determining the angles of yaw, pitch, and roll that define how the head is oriented in space. These three angles are commonly used to describe head orientation in images. Here, the terms “pose” and “orientation” are often used interchangeably. Recently, deep learning-based pose estimation models have advanced, enabling more precise estimation of head direction. Traditionally, head pose estimation involved first locating facial landmarks and then calculating the head orientation from these points [19,20]. However, this approach suffers from reduced accuracy when landmarks are occluded or poorly visible. To address these limitations, newer methods now predict the head pose directly from the image (before landmark detection) and then use the estimated pose information to locate facial landmarks more accurately—examples include FSA-Net [21] and HopeNet [22]. With these advances, it is now possible to accurately determine the orientation of a head in images, supporting objective analysis of emotional changes and stress responses.

There have been no previous studies that conducted deep learning-based pose estimation specifically focused on the orientation of cattle heads and ears for the objective assessment of stress. The present study applies Mask R-CNN for object detection and FSA-Net for pose estimation to detect and analyze head and ear orientation behaviors in cattle in a quantitative manner. Mask R-CNN is effective at accurately detecting cattle heads and ears, while FSA-Net can finely estimate their pose, offering significant technical advantages for animal behavior research. By leveraging these AI technologies, this study aims to provide objective, non-invasive, and long-term pose analysis methods for evaluating stress and pain in animals.

## 2. Materials and Methods

### 2.1. Overview of Our Framework

**Figure 1:**
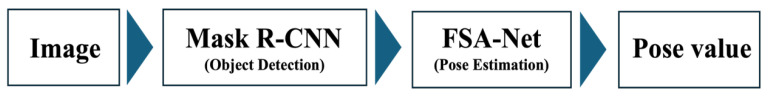
Overview of the Framework. A schematic illustration of the proposed system for cattle head and ear detection and pose estimation, highlighting the two main steps: object detection and pose estimation.

**Figure 2:**
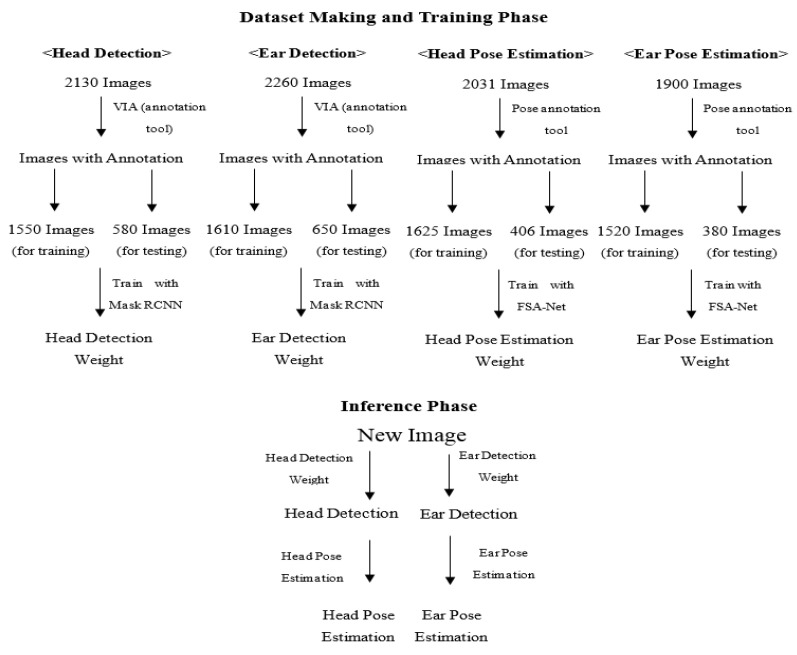
Dataset Composition and Model Workflow. Visual representation of the dataset organization (head and left ear detection and pose estimation groups), along with the workflow of the trained models.

Our system is designed to detect and estimate the 3D pose of a cow’s head and left ear from images. The process is split into two main steps for each part: detection and pose estimation (Figure 1). For the detection stage, we found that, while tools such as OpenCV are highly effective for human face detection, existing libraries lack support for animal-specific features. As a result, we created datasets for both the head and left ear and trained them using Mask R-CNN [11] for detection. The training process saved important parameters as weights, which allow us to find these parts in new images. For pose estimation, we also constructed datasets and trained them with FSA-Net [21], resulting in separate weights for head and ear pose. Overall, we prepared four datasets and trained them with the appropriate algorithms. Now, using the trained models, we can accurately locate the cow’s head or ear and determine its orientation in any new image (Figure 2).

### 2.2. Image Acquisition and Dataset Composition

For this research, RGB images were gathered from 88 Japanese Black cattle of all ages, ranging from calves to mature adults (Agriculture of Miyazaki University, Japan). All photographs were taken opportunistically, rather than with a fixed camera, with an iPhone X camera in natural daylight, and multiple viewing angles were employed to create a comprehensive and varied image collection. Ethical approval for the study was secured from the Animal Ethics Committee of Miyazaki University (approval number: 2021-043). Of the 88 cattle, images from 67 individuals were used for training and validation, and those from the remaining 21 were used for testing. The images for detection were organized into two primary groups according to anatomical features: head and left ear. The head detection group contained 1550 images for training and validation and 580 for testing, while the left ear detection group included 1610 training and validation images and 650 test images. Likewise, the images for pose estimation were split into head and left ear categories. The head pose estimation group had 1625 training and validation images and 406 test images, and the left ear pose estimation group was composed of 1520 training and validation images and 380 test images (Figure 2).

### 2.3. Object Detection: Annotation and Dataset Construction

**Figure 3:**
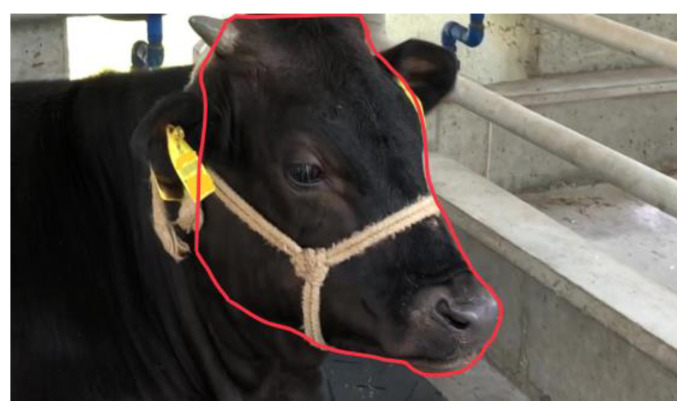
Object Detection Annotation Process Using VIA. Demonstration of the image annotation process for object detection (cattle head), showing the use of the VGG Image Annotator (VIA) to label regions of interest (ROIs) in cattle images (red line). The image shown is of an animal actually used in this study.

An Object detection dataset consists of images and their corresponding annotations. Image annotation is the process of labeling specific regions or objects within an image to make them recognizable to machine learning models. This is typically performed to provide ground-truth data for training and validation, allowing the model to learn from accurately marked examples. Image annotation was performed by an expert using the VIA (VGG Image Annotator, Figure 3) [23]. For the head dataset, the region of interest (ROI) was defined as the area from the top of the head to the muzzle, including the lower jaw, and reaching caudally to the caudal boundary of the mandible, while excluding the neck. For the left ear dataset, the ROI included the entire pinna, including both the inner and outer surfaces, the base of the ear where it attaches to the head, and the tip of the auricle. This ensured that all visible anatomical features of the left ear were fully included in the annotation. Through this process, two object detection datasets were created: one for cattle head, consisting of 2130 images with corresponding annotations (1550 for training and validation and 580 for test), and another for cattle left ear, comprising 2260 images with annotations (1610 for training and validation and 650 for test).

### 2.4. Object Detection: Model Training

For model training on the dataset, the Mask R-CNN framework [11] was used, with ResNet-101 as the backbone. The model was pre-trained on the COCO dataset [24], which contains over 330,000 images, including a large number of animal images. Transfer learning was employed to leverage the pre-trained weights, with the first 80 layers of the backbone frozen to accelerate training and prevent overfitting. The final dense layers were fine-tuned to better fit the morphological characteristics of the cattle’s head and left ear. Model training was performed on an AWS p2.xlarge instance equipped with a single NVIDIA Tesla K80 GPU (12GB VRAM) and 61 GiB RAM. Training hyperparameters were as follows: batch size of 1, learning rate of 0.001, and 100 epochs for Mask R-CNN. Data augmentation included random rotation and flipping. The system achieved an inference speed of 5 frames per second (FPS) on 640 × 480 pixel images, using the same environment as the training setup (Table 1).

### 2.5. Pose Estimation: 3D Pose Annotation and Dataset Construction

**Figure 4:**
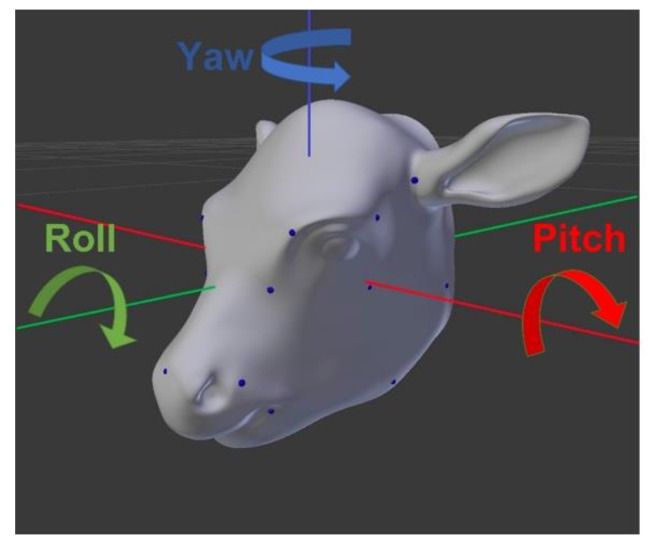
Vectors for Pose Estimation. The blue dots indicate the 21 landmarks, the green line represents the x-axis, the red line represents the y-axis, and the blue line represents the z-axis.

The pose estimation dataset included each image annotated with a set of 3D orientation vectors—specifically, yaw, pitch, and roll values (Figure 4). These vectors describe the rotation of an object around three perpendicular axes: yaw corresponds to rotation around the vertical axis (left and right), pitch to rotation around the side-to-side axis (up and down), and roll to rotation around the front-to-back axis (tilting side to side).

**Figure 5:**
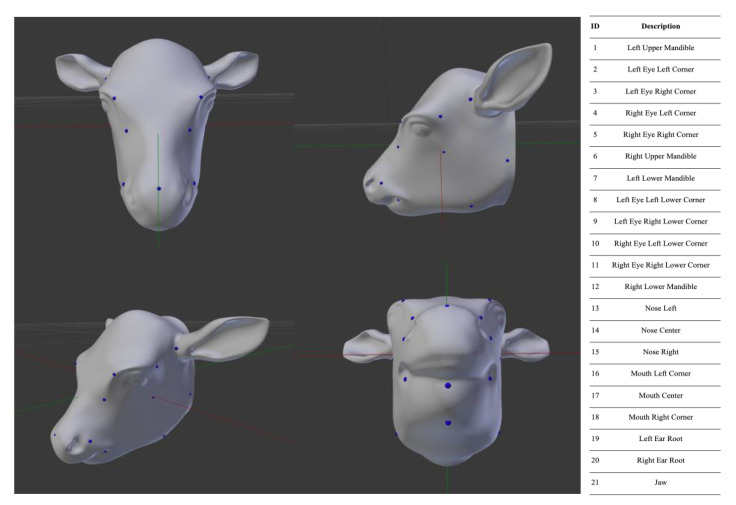
Anatomical Landmarks on the Cow’s Head Model in Blender. A 3D model of the cow’s head created in Blender (**left**), with 21 anatomical landmarks (**right**) positioned at key locations for accurate 3D pose estimation.

To generate these pose annotations, we used the AFLW (Annotated Facial Landmarks in the Wild) tool [25], which is based on the Posit algorithm [26]. The Posit algorithm is a classical approach for estimating the 3D pose of an object from a single 2D image by leveraging known correspondences between 3D model points and their 2D projections. The AFLW tool operates through a C++ interface with an SQL database. The process to calculate the pose is as follows: (1) The process began by creating custom 3D models of the cow’s head and left ear using Blender. (2) Within Blender, we placed anatomical landmarks on each model and recorded their 3D coordinates. For the cow’s head, we positioned 21 landmarks at key locations, following the structure of the AFLW used for the human face. However, because of anatomical differences between the human face and the cow head, a perfect one-to-one mapping is not always possible. To address this, we mapped as many landmarks as possible based on anatomical similarity and spatial correspondence. For regions where a direct match was not possible, the closest functional or spatially analogous point on the cow head was selected (Table 2). Accordingly, the 21 landmarks defined on the cow head include the back and lower center of the mandible, four points around each eye (upper and lower, left and right), three points across the external nose (left, center, and right), the base of the ear attachment, three points along the mouth (left, center, and right), and the lowest point of the jaw (Figure 5).

This table summarizes the correspondence between the 21 facial landmarks defined in the AFLW human face dataset and the 21 anatomical landmarks established on the cow head model. The mapping was determined based on anatomical similarity and spatial correspondence, enabling the adaptation of human head pose estimation methods to cattle. Where a direct one-to-one match was not possible due to structural differences, the closest analogous region on the cow head was selected.

**Figure 6:**
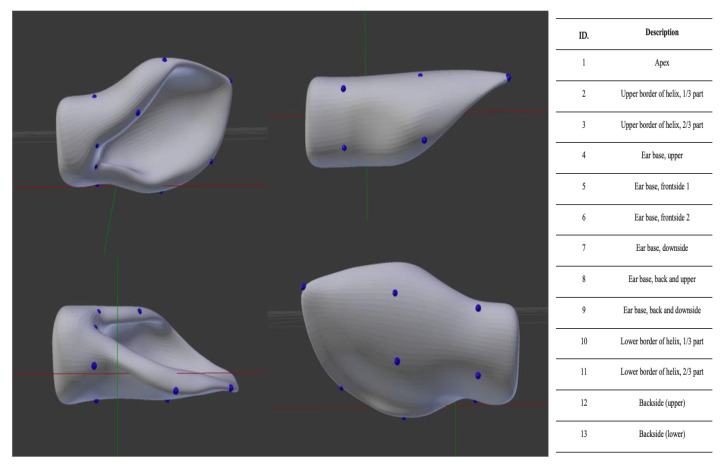
Anatomical Landmarks on the Cow’s Left Ear Model in Blender. A 3D model of the cow’s left ear was created in Blender (**left**), with 13 landmarks (**right**) distributed from the apex to the base to ensure precise 3D pose estimation.

Unlike the human face, there is no standardized set of anatomical landmarks for the ear in cattle. Therefore, in this study, we defined 13 evenly distributed landmarks from the apex to the base to ensure accurate 3D pose estimation (Figure 6).

**Figure 7:**
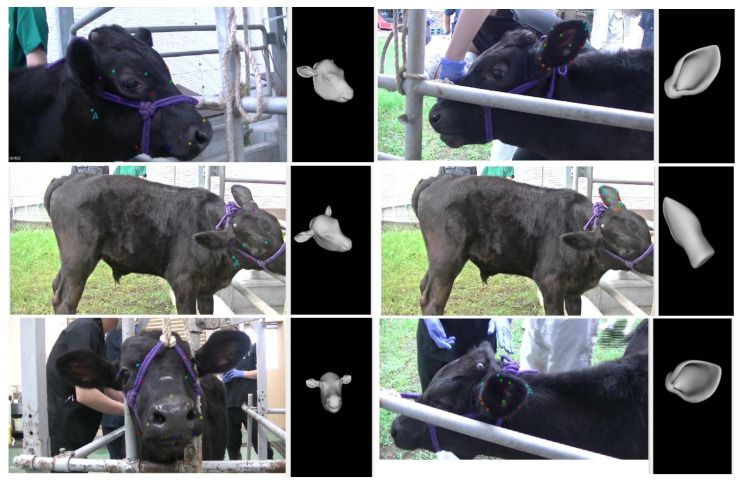
Landmark Matching and Pose Calculation Using the AFLW Tool. Visualization of the process where a human annotator selects corresponding landmarks on 2D images within the AFLW program, enabling the Posit algorithm to calculate the precise 3D orientation of the head or ear.

(3) These models were then imported into the AFLW tool, where a human annotator selected the corresponding landmarks on the 2D images. (4) By matching the 3D model landmarks to those identified in the images, the Posit algorithm calculated the precise orientation of the head or ear, resulting in yaw, pitch, and roll values for each image (Figure 7). Ultimately, this process produced two comprehensive pose estimation datasets: one for the cow’s head, consisting of 2031 images along with their corresponding orientation vectors, and one for the cow’s left ear, containing 1900 images and their associated orientation vectors. The constructed dataset was divided into three parts: 60% for training, 20% for validation, and 20% for testing.

### 2.6. Pose Estimation: Model Training

For the FSA-Net architecture, several model variants exist depending on the choice of feature aggregation method and scoring function. During image processing, feature maps are generated, and the aggregation method compresses these into a smaller set of highly representative features. Common aggregation approaches include capsule networks and NetVLAD; in our study, we adopted the capsule aggregation method, following the approach detailed in the original FSA paper [21]. While the aggregation method treats input features as an unordered collection (a “bag of features”), the scoring function organizes these features spatially. The FSA-Net framework supports several scoring functions, as described in previous research: 1 × 1 convolution, variance-based, and uniform scoring. The 1 × 1 convolution approach uses a learnable convolution kernel to assign importance to each feature, which can be expressed as

$$\Phi \left(u\right)=\sigma \;(w\;\cdot \;\;u)$$

where $u={(u}_{1},\cdot \cdot \cdot ,u_{c})$ is a pixel-level feature, Φ(u) is a scoring function, σ is the sigmoid function, and w is the learnable convolution kernel. In the variance-based scoring function, features are selected based on their variability, calculated as

$$\Phi \left(u\right)=\sum_{i=1\;}^{c}{\left(u_{i}-\mu \right)}^{2}\;(\mathrm{deviation}\mathrm{of}\mathrm{the}\mathrm{pixel}-\mathrm{level}\mathrm{feature})$$

where $\mu =\frac{1}{c}\sum_{i=1}^{c\;}u_{i}$ (average of the pixel-level feature). The uniform scoring function treats all features equally, assigning a constant value of 1 to each.

$$\Phi \left(u\right)=1$$

Our datasets were trained using each of these scoring function options, resulting in distinct model weights for each configuration. This approach allowed us to evaluate the impact of different scoring strategies on model performance. Model training was performed on a system with a 2.3 GHz 8-core Intel Core i9 CPU (Intel, Santa Clara, California, United States), AMD Radeon Pro 5500M (8GB) (AMD, Santa Clara, California, United States), 32GB RAM (SK Hynix, Seoul, South Korea). Training hyperparameters for FSA-Net were as follows: batch size of 16, learning rate of 0.001, and 90 epochs. The inference speed was 17.32 ms per image for 64 × 64 pixel inputs, using the same environment as the training setup (Table 3).

### 2.7. Performance Evaluation

To evaluate the accuracy of our algorithms, we used two main metrics suited to each task: mean average precision (mAP) for object detection and mean absolute error (MAE) for pose estimation. For object detection, mAP is a widely accepted metric that assesses how well the model identifies and localizes objects within images. The calculation is based on precision and recall, which are determined using the intersection over union (IoU) between predicted and ground-truth bounding boxes. The IoU threshold was set at 0.5, meaning a detection is considered correct only if the overlap between predicted and true bounding boxes exceeds 50%. Precision is the ratio of correct detections to all detections made by the model, while recall is the ratio of correct detections to all actual objects present in the image. For example, if there are five actual objects and the model detects three—with two correct—the precision is 66.7% (2/3) and the recall is 40% (2/5). Since only one object was present and predicted per image in this study, precision and recall values were calculated at this fixed threshold, and the mAP was reported as the representative performance metric. Higher mAP values, ranging from 0 to 1, indicate better detection performance. For pose estimation, we used MAE to measure the difference between the algorithm’s predicted pose (yaw, pitch, roll) and the ground-truth pose labeled by a human expert. MAE is calculated as the average absolute difference between predicted and true pose vectors across all images. The formula is:

$$MAE=\frac{1}{N}\sum_{n=1}^{N}∥{ỹ}_{n}-{\;y}_{n}∥$$

where *N* is the number of images, $y_{n}$ is the predicted pose vector, and ${ỹ}_{n}$ is the ground-truth pose vector. Lower MAE values indicate higher pose estimation accuracy. To contextualize our results, we compared the MAE for cow pose estimation with established benchmarks from human head pose estimation [21] using the AFLW dataset. This dual-metric approach ensures a comprehensive evaluation of both detection and pose estimation performance in our study.

## 3. Results

The object detection algorithm, based on Mask R-CNN, achieved an mAP of 0.79 for cattle head detection and 0.71 for left ear detection, using an IoU threshold of 0.5. In the validation of head detection, the model demonstrated robust performance across a range of head detections and orientations. However, during the inference phase, the model occasionally failed to detect the head when it was tilted almost completely backward. For left ear detection, the model consistently identified the target region in most images, though with a slightly lower mAP compared to head detection.

For pose estimation, the FSA-Net architecture was trained with three different scoring functions: without fine-grained feature mapping, with 1 × 1 convolution, and with variance-based scoring. The mean absolute error (MAE) for the head pose estimation ranged around 8 across all three scoring functions, while the MAE for left ear pose estimation was close to 9 (Table 4). These results were obtained using capsule aggregation for feature extraction and were evaluated against ground-truth pose annotations generated by human experts.

**Figure 8:**
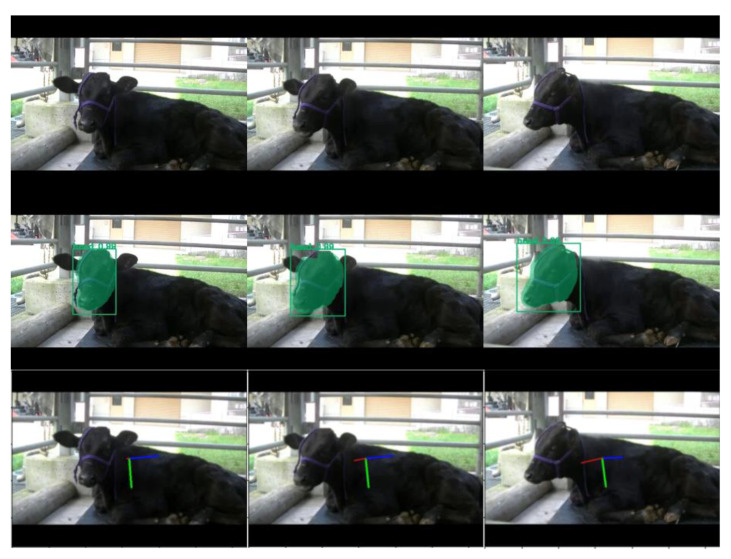
Sample results of head detection and pose estimation using the proposed method. The images illustrate a sequence of cattle head motion across multiple frames. In the second row, green bounding boxes and segmentation masks indicate the detected head regions. The third row displays the output of head pose estimation: a red line represents the direction the subject is facing (yaw), a green line indicates the downward direction (pitch), and a blue line corresponds to the sideways direction (roll).

**Figure 9:**
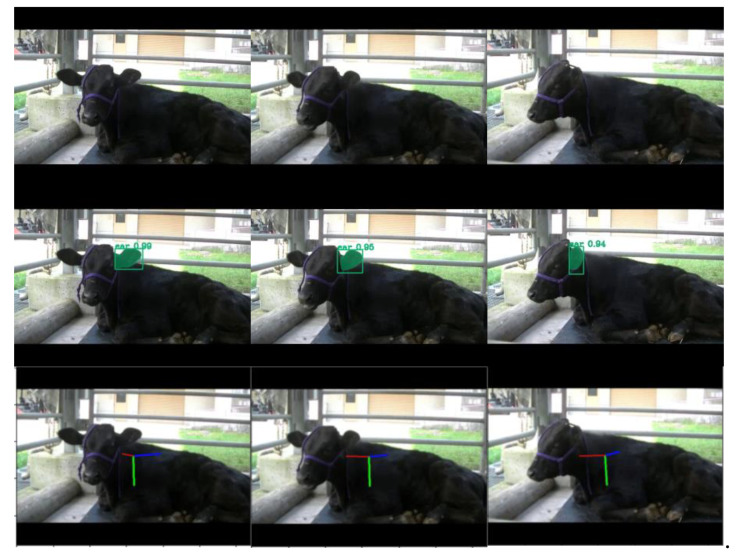
Sample results of ear detection and pose estimation using the proposed method. The images illustrate a sequence of cattle ear motion across multiple frames. In the second row, green bounding boxes and segmentation masks indicate the detected ear regions. The third row displays the output of ear pose estimation: a red line represents the direction the subject is facing (yaw), a green line indicates the downward direction (pitch), and a blue line corresponds to the sideways direction (roll).

The trained models were applied to new images excluded from the original training/validation datasets. Object detection outputs, including bounding boxes and segmentation masks, were overlaid on the images alongside pose vectors representing yaw, pitch, and roll angles (Figure 8 and Figure 9). These visualizations confirmed the detection of cattle heads and left ears across diverse orientations, with pose vectors quantitatively representing their spatial alignment.

## 4. Discussion

The AI-based system proposed in this study reliably detects and estimates the orientation of cattle heads and ears, significantly enhancing the objectivity and feasibility of long-term behavioral stress assessment. Traditionally, blood cortisol concentration has been regarded as the gold standard for evaluating stress levels in animals, with numerous studies utilizing it to assess stress related to castration [27], heat stress [28], and calf weaning [29]. However, blood cortisol measurement requires invasive blood sampling, which poses animal welfare concerns, and the process is costly, time-consuming, and unsuitable for real-time monitoring. Alternative approaches such as salivary [30], fecal [31], and hair cortisol [32] measurements have been explored to evaluate acute and chronic stress, but these methods still face challenges related to cost, and delayed analysis. In response to these limitations, behavioral and visual observations have been widely used to assess stress and pain in animals [2,3,4]. Nevertheless, such observations are often subjective, varying between observers, and long-term monitoring is difficult, which compromises the reliability and consistency of stress evaluation. Our study addresses these challenges by leveraging advanced deep learning techniques—Mask R-CNN [11] for precise detection and FSA-Net [21] for accurate 3D pose estimation (yaw, pitch, roll)—to quantitatively analyze the head and ear orientations of cattle. The system demonstrated high detection accuracy (mAP between 0.71 and 0.79) and pose estimation precision (MAE around 8 to 9), confirming its robustness across various poses. This technological advancement holds significant implications for animal welfare research and practical applications. The AI-based approach is non-invasive, cost-effective, and capable of real-time monitoring, thus overcoming the inherent drawbacks of cortisol-based methods. Furthermore, by enabling the long-term collection of objective and quantitative data on animal behavior, it mitigates observer bias and enables systematic stress management.

To evaluate the accuracy of the object detection algorithm in this study, we used mAP as the primary metric. mAP is a widely accepted standard in object detection tasks, as it summarizes the model’s ability to correctly identify and localize objects across the entire dataset. The calculation of mAP is based on the integration of the precision-recall (PR) curve, which itself is determined by the relationship between precision (the proportion of correct detections among all detections) and recall (the proportion of correct detections among all actual objects present in the image), using a fixed IoU threshold of 0.5. However, in the specific context of this study, the use of PR curves as a performance metric has inherent limitations. Since the model was designed to detect at most one object (either the head or the left ear of a cow) per image, and each image contained only a single target object, adjusting the confidence threshold does not result in multiple predictions per image. Instead, the threshold only determines whether the single predicted object is accepted or rejected. Consequently, the precision and recall values for each image can only take on extreme values, such as (1, 1) or (0, 0), depending on whether the prediction passes the threshold. Even when averaged across multiple images, the resulting PR curve is essentially a straight line parallel to the x-axis or a single point, providing no additional informative value beyond what is already summarized by the mAP. For these reasons, it was deemed appropriate in this study to present only the mAP value instead of PR curves. However, in the future, it will be necessary to detect multiple objects simultaneously within a single image. For example, in addition to pose estimation using the detection of the cow’s head or left ear as conducted in this study, comprehensive analysis of various physiological signals—such as analyzing breathing patterns by detecting the nostrils [12], or monitoring changes in body temperature by detecting the eyes or muzzle [13]—will require the simultaneous detection of multiple objects in one image. If only one object is detected per image, the same image must be processed multiple times for different targets, which complicates the analysis process and reduces model efficiency. Therefore, it would be desirable to adopt models capable of detecting multiple objects in a single inference and to employ appropriate evaluation metrics, such as class-wise PR curves, for such models.

**Figure 10:**
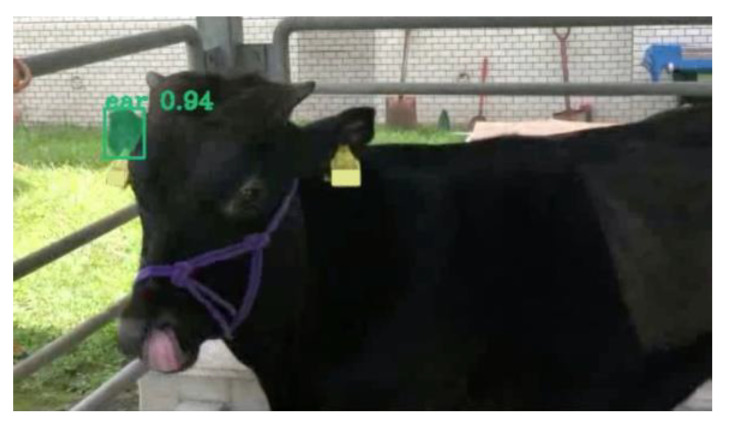
Example of Ear Detection Failure. The image shows how the AI system can mistake the left and right ears of cattle due to their similar appearance. Here, the right ear is detected (green box), but the similarity and position of both ears make it difficult for the system to distinguish between them.

As a prior study relevant to this research, FSA-Net [21], proposed for the human face domain, is a lightweight model that directly estimates 3D head pose (yaw, pitch, and roll) from a single RGB image without relying on facial landmarks. It is characterized by high accuracy and low memory consumption. Unlike traditional landmark-based pose estimation methods [19,20], which tend to be computationally expensive and involve large models, FSA-Net learns fine-grained spatial feature structures to enable efficient and precise pose regression. In this study, we adapted the human-face FSA-Net model through transfer learning to estimate the head and ear poses of cattle. By integrating Mask R-CNN for object detection, we developed a 3D pose estimation system specialized for animal behavior research. While prior human face studies focus on estimating the pose of the entire face, our approach targets specific regions—the cattle’s head and left ear—that are critical for stress and behavior analysis. This focus enables non-invasive, long-term, and quantitative monitoring, distinguishing our work from previous research. Our AI-based system achieved a mean absolute error (MAE) of approximately 8–9 degrees in estimating cattle head and ear poses. Although this accuracy is lower than the 5 to 6 degrees MAE reported for the original human-face FSA-Net, it remains a reasonable performance considering the unique challenges posed by animal morphology, dataset size, and environmental conditions. Specifically, the cattle’s ears present difficulties due to their simpler shape and coloration compared to human faces, and the strong similarity between left and right ears often causes confusion during object detection (Figure 10), especially in lateral views. This likely contributed to occasional misdetections or unclear boundaries in the Mask R-CNN stage, subsequently reducing pose estimation accuracy. Moreover, our dataset was collected under natural lighting conditions using an iPhone X, and the predominantly black coat of Japanese Black cattle makes object detection and pose estimation more susceptible to variations in illumination, shadows, and complex backgrounds. In contrast, human face datasets like AFLW [25] contain approximately 26,000 images encompassing diverse ethnicities, lighting, angles, and expressions, enabling models to learn from a wide variety of poses. Our cattle dataset, by comparison, includes around 2000 images with more limited pose and environmental diversity, which may have led to data imbalance and reduced generalization capability. Despite these limitations, the achieved MAE is sufficiently practical for quantitative pose estimation in animal behavior analysis. Our results demonstrate that the technical strengths of FSA-Net for human head pose estimation can be effectively transferred and adapted to the domain of animal behavior research.

Given the relatively low accuracy in pose estimation mentioned above, future research should focus on building large-scale datasets that include a variety of cattle breeds and environmental conditions to enhance the robustness of the model. While the current study utilized a dataset limited to Japanese Black cattle, real-world farm environments feature diverse breeds such as Holstein and Angus, as well as a wide range of settings, including indoor and outdoor housing, grazing pastures, varying lighting, and camera angles. By collecting data that reflects these complex conditions, the model’s generalization performance can be improved, enabling it to operate reliably across different scenarios. Additionally, to improve the accuracy of cattle head and ear pose estimation, future work should use modern, lightweight deep learning models designed for real-time processing. A multi-task neural network (MNN) [33] combines an encoder-decoder structure with residual blocks and skip connections. In this model, the head pose estimation task is placed at the end of the encoder, where it can use global features from the whole image, while tasks like landmark detection and visibility are handled at the end of the decoder, where more detailed, local information is available. This approach helps the model to learn useful features for both pose and other related tasks at the same time, which improves overall accuracy. By using this kind of advanced architecture, it is possible to build a system that can quickly and accurately analyze the poses of cattle heads and ears in real time. Furthermore, because this study enables long-term quantitative analysis of cattle head and ear poses, it allows for a wide range of quantitative and detailed analysis of pose patterns [34]. Based on these capabilities, it is necessary to perform comparative analyses between these pose patterns and serum cortisol levels—the gold standard for stress assessment—across various environments. For example, by analyzing the correlation between head/ear pose patterns and blood cortisol concentrations during stress-inducing events such as passage through narrow corridors or unfamiliar human contact, it is possible to determine whether specific pose patterns are statistically associated with stress responses. Such analyses can provide important insights into the mechanisms linking animal behavior and physiology. Moreover, assessing animal stress and pain based solely on head and ear poses has its limitations. Therefore, building a multimodal AI system that goes beyond just head and ear pose estimation is important. By integrating open-source tools like DeepLabCut [14] and LabGym [17] to analyze behavioral patterns, along with infrared cameras for monitoring breathing [12] and body temperature [13], and accelerometers for detecting rumination [35], we can obtain a more accurate and holistic picture of animal health and stress. Finally, since the primary objective of this system is to objectively evaluate animal behavior for stress and pain assessment in real farm settings, it is essential to develop a user-friendly interface for farm managers and veterinarians. By providing practical features such as real-time alert systems and data dashboards that visualize individual health trends and stress indices, the system’s applicability in actual farm environments can be enhanced. These advancements are expected to make significant contributions to the improvement of animal welfare and the sustainability of livestock farming.

Recently, there has been significant progress in precision livestock farming through the use of lightweight deep learning models and embedded systems capable of real-time operation. Notably, lightweight variants of the YOLO family, such as YOLOv4-tiny [36], have been widely adopted for their computational efficiency and fast inference speed, enabling real-time object detection and behavioral analysis in various environments, including on-farm CCTV, smartphones, and edge devices [37]. These models are characterized by low memory usage and can be easily deployed on mobile or embedded systems without the need for high-end GPUs. Several recent studies have successfully applied YOLO-based models for real-time monitoring of livestock—such as cattle [38] and sheep [39]—to detect abnormal behaviors, disease symptoms, and individual identification. The pose estimation system developed in this study, based on Mask R-CNN and FSA-Net, also has the potential for optimization and deployment on mobile or edge devices. In particular, FSA-Net is advantageous for real-time processing due to its lower memory requirements compared to traditional landmark-based methods and its ability to estimate 3D pose from a single RGB image. Future research should include direct comparisons with lightweight YOLO variants in terms of performance and efficiency, validation of real-time operation in mobile and embedded environments, and evaluation of applicability across various hardware platforms. Such efforts will further advance the development of automated, real-time welfare monitoring systems for practical use in livestock farming.

The AI-based pose estimation system developed in this study is designed to enable long-term, objective, and automated monitoring of cattle head and ear positions in real farm environments. By integrating with on-farm CCTV or smart cameras, the system can continuously detect and record behavioral changes—such as responses to unfamiliar humans or movement through narrow corridors—in real time. This approach offers a non-invasive and objective alternative to traditional methods that rely on subjective observation or invasive physiological measurements like blood cortisol sampling, thereby allowing for continuous welfare assessment without imposing additional stress on the animals. In practical terms, the system provides several key benefits: real-time monitoring enables early detection of stress or abnormal behaviors, supporting timely intervention and disease prevention; quantitative assessment is possible using only video data, eliminating the need for repeated blood sampling or sensor attachment; and visualization of individual behavioral data facilitates efficient farm management by allowing farm personnel to easily track health trends. Furthermore, the accumulation of large-scale behavioral datasets will support future research on correlations with established welfare indicators and inform evidence-based policy development. It should be noted, however, that this study primarily focused on evaluating the accuracy of pose estimation. Further research is required to statistically validate the associations between behavioral data obtained through this system and physiological welfare indicators such as blood cortisol levels or disease incidence. With continued validation across diverse breeds and environments, and the development of user-friendly interfaces, the system is expected to make a substantial contribution to animal welfare monitoring and the sustainability of livestock farming.

## 5. Conclusions

This study successfully developed an AI-based system for non-invasive detection and pose estimation of cattle heads and ears, enabling long-term and quantitative assessment of stress and pain in cattle. By leveraging advanced deep learning models—Mask R-CNN for detection and FSA-Net for pose estimation—the system demonstrated robust performance in identifying and analyzing cattle head and ear orientations under natural conditions. Moreover, the system is designed with real-time implementation in mind, making it suitable for deployment on farm-based CCTV or smart camera platforms. This capability allows for continuous, objective assessment of cattle welfare in practical farm settings, supporting early detection of stress or abnormal behaviors and facilitating timely intervention. By providing reliable, real-time behavioral insights without the need for invasive procedures or manual observation, this technology has the potential to transform animal welfare monitoring and promote more sustainable livestock management. Future work should focus on further optimizing the model for embedded and mobile devices, expanding the dataset to encompass a wider range of breeds and environments, and validating the system’s effectiveness through longitudinal studies that correlate pose patterns with established physiological welfare indicators.

## Figures and Tables

**Table 1:** Summary of dataset composition, key hyperparameters, and inference speed for head and left ear detection tasks. # indicates the number of images.

Task	#Training Images	#Validation Images	Annotation Type	Model (Backbone)	Batch Size	Learning Rate (Epochs)	Data Augmentation	Inference Speed
Head Detection	1170	380	Bounding Box	Mask R-CNN (ResNet-101)	1	0.001 (100)	Rotation, Flipping	5 FPS (640 × 480 pixel)
Left Ear Detection	1210	400						

**Table 2:** Mapping between AFLW (human face) 21 landmarks and cow head 21 anatomical landmarks used in this study.

	AFLW Landmarks (Human)	Cattle Head Landmarks	Mapping Type/Notes
1	Left Brow Left Corner	Left Upper Mandible	Closest anatomical region
2	Left Brow Center	Left Eye Left Corner	Upper left eye region
3	Left Brow Right Corner	Left Eye Right Corner	Upper right eye region
4	Right Brow Left Corner	Right Eye Left Corner	Upper left of right eye
5	Right Brow Center	Right Eye Right Corner	Upper right of right eye
6	Right Brow Right Corner	Right Upper Mandible	Closest anatomical region
7	Left Eye Left Corner	Left Lower Mandible	Lower left eye region
8	Left Eye Center	Left Eye Right Lower Corner	Lower left of left eye
9	Left Eye Right Corner	Left Eye Right Lower Corner	Lower right of left eye
10	Right Eye Left Corner	Right Eye Left Lower Corner	Lower left of right eye
11	Right Eye Center	Right Eye Right Lower Corner	Lower right of right eye
12	Right Eye Right Corner	Right Lower Mandible	Lower right face region
13	Nose Left	Nose Left	1:1 mapping
14	Nose Center	Nose Center	1:1 mapping
15	Nose Right	Nose Right	1:1 mapping
16	Mouth Left Corner	Mouth Left Corner	1:1 mapping
17	Mouth Center	Mouth Center	1:1 mapping
18	Mouth Right Corner	Mouth Right Corner	1:1 mapping
19	Left Ear Lobe	Left Ear Root	Closest to ear base
20	Right Ear Lobe	Right Ear Root	Closest to ear base
21	Chin Center	Jaw	1:1 mapping

**Table 3:** Summary of dataset composition, key hyperparameters, and inference speed for head and ear pose estimation tasks. # indicates the number of images.

Task	#Training Images	#Validation Images	Annotation Type	Model	Backbone	Batch Size	Learning Rate	Epochs	Inference Speed
Head Detection	1225	400	3D Pose	FSA-Net	Capsule	16	0.001	90	17.32 ms/image (64 × 64 pixel)
Left Ear Detection	1140	380							

**Table 4:** Pose estimation performance (MAE) across datasets and scoring functions. The FSA-Net architecture was evaluated across three datasets: the human face dataset (AFLW), the cow head pose dataset, and the cow ear pose dataset. For all experiments, capsule aggregation was used for feature extraction, and three pixelwise scoring functions were tested: without fine-grained feature mapping (w/o), with 1 × 1 convolution (1 × 1), and variance-based scoring (var). The mean absolute error (MAE) for each configuration is summarized in Table 4.

Testing Set	AFLW			Cow Head Pose Dataset			Cow Ear Pose Dataset		
Method	FSA-Net								
Aggregation	Capsule								
Pixelwise Scoring	w/o	1 × 1	var	w/o	1 × 1	var	w/o	1 × 1	var
MAE	5.75	5.25	5.36	7.95	8.09	7.63	9.78	9.60	8.66

## Data Availability

The datasets generated for this study are available on request to the corresponding author.
